# Application of Immediate Dentoalveolar Restoration in Alveolus Compromised with Loss of Immediate Implant in Esthetic Area

**DOI:** 10.1155/2018/1672170

**Published:** 2018-06-21

**Authors:** Rafael de Lima Franceschi, Luciano Drechsel, Guenther Schuldt Filho

**Affiliations:** ^1^Brasilian Association of Dental Surgeons, Curitiba, PR, Brazil; ^2^Dental Institute of the Americas, Balneário Camboriú, SC, Brazil; ^3^São Leopoldo Mandic University, Curitiba, PR, Brazil; ^4^Brazilian Dental Association (ABO), Ponta Grossa, PR, Brazil; ^5^Federal University of Santa Catarina, Florianópolis, SC, Brazil

## Abstract

In the reported clinical case, the immediate dentoalveolar restoration (IDR) technique was applied to reconstruct the buccal bone wall, with autogenous graft of the maxillary tuberosity, which had been lost due to a root fracture, and to provide the necessary bone substrate for the installation of an implant and its provisioning. One of the greatest risks inherent in the survival of immediate implants is the maintenance of their stability during the healing period. In this case, due to a mechanical trauma in sports activity in the first postoperative month, there was a total failure in the osseointegration process, confirmed by tomographic examination of both the implant and the bone graft. The deleterious effects of this accident were compensated with a new approach and reapplication of IDR technique using a smaller-diameter implant and with conical macrogeometry in conjunction with the new bone reconstruction under the same compromised alveolus; associated, after the period of osseointegration, with the maneuvers of volume increase of the gingival tissue by subepithelial connective tissue graft. The tomographic result demonstrated the success of the surgical procedures, and the clinical/photographic analysis obtained showed the stability of the gingival margin without compromising the esthetic result of the prosthetic restoration.

## 1. Introduction

Esthetic dentistry has currently reached a level of excellence that no longer allows teeth subject to exodontia for reasons such as trauma and bacterial infections, resulting in prosthetic works that do not adequately mimic homologous teeth and the surrounding gingival tissues. The challenge of removing a dental element and facing the events that occur on the protective and sustaining tissues requires deep knowledge in the areas of periodontics, surgery, and dental prosthesis [1].

Partial or total loss of the vestibular bone wall potentiates the deleterious effects on the periodontal tissues that occur in a postextraction alveolus and indicate the need for preservation and simultaneous alveolar reconstruction to the exodontia, allowing the stability of these tissues and enhancing their clinical predictability [2]. The possibility of installing an immediate implant and its adequate provisioning are is a factor that contributes to the maintenance of esthetics and peri-implant health, and when associated with the autogenous graft, the osteogenic, osteoinductive, and osteoconductive capacity of this material results in a more rapid vascularization and incorporation, with no immune response, being the gold standard of regenerative procedures [3].

Immediate loading, even in infraocclusion, to which immediate implants are installed postexodontia in esthetic areas, increases the risks of postoperative failures and complications [4]. If the loss of a dental element is already bad, an immediate implant is even worse because the bone bed has already been milled, and failure in the osseointegration process results in a rapid formation of granulation tissue; alteration of local irrigation; formation of areas of necrosis, in short, a severe collapse of the peri-implant tissues with increased bone remodeling; and alteration in the vestibular gingival margin, compromising the esthetic result of the procedure [5, 6].

The immediate dentoalveolar restoration (IDR) technique aims at regenerating, via the autogenous bone of the maxillary tuberosity, the postextracting alveolus that has one or more compromised bone walls, allowing the installation of an immediate implant and its provisioning. In the situation of loss of one immediate implant with total loss of the vestibular bone wall, IDR would be a viable tool to repair the alveolus, creating a clinical situation that allows the installation of a new implant and its prosthesis, immediately recovering the esthetics of the patient who can subsequently receive regenerative procedures aimed at maintaining or improving the level and contours of the peri-implant soft tissues, which are critical for esthetics and long-term success [7].

## 2. Case Report

**Figure 1:**
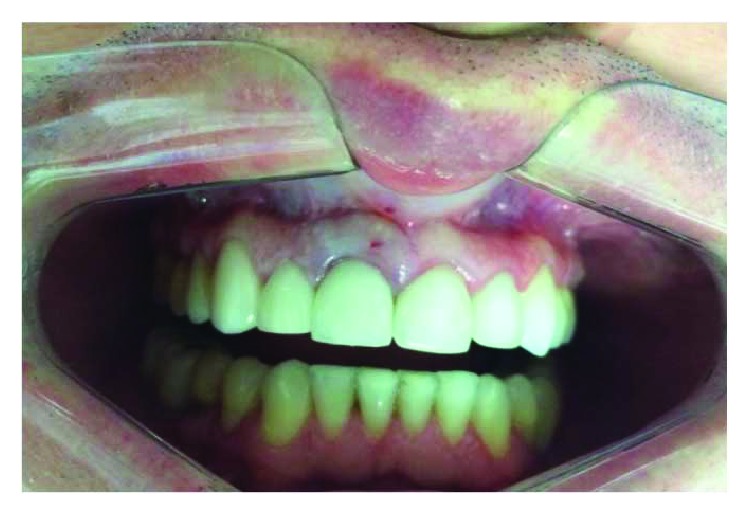
Presence of ceramic restorations in the upper anterior teeth with swollen gingival area on the vestibular face of element 11.

A male patient, thirty-five years of age, attended the clinic for care after reporting intense pain during chewing in which he reported hearing a fractured tooth 11 and its subsequent mobility. He received a ceramic crown (metal-free) rehabilitation on the upper teeth two years ago. The clinical examination revealed that the gingival biotype was thick, there was a small active fistula on the vestibular surface, and the depth of probing in this area was 7 mm, with mobility of the crown in the vestibular-palatine direction (Figure 1).

**Figure 2:**
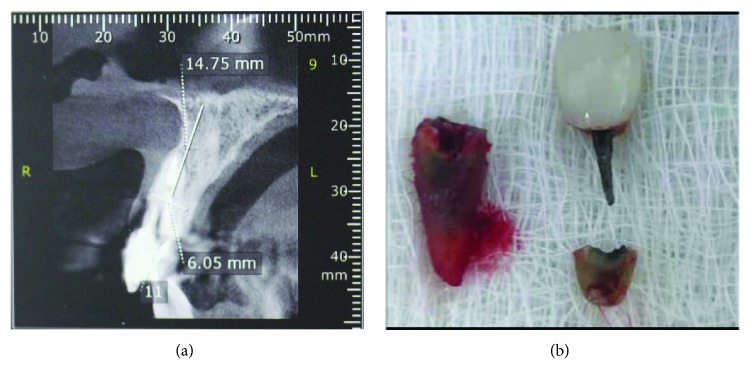
Root fracture and its subsequent bacterial contamination caused the vestibular wall to become impaired. (a) Tomography demonstrating the loss of vestibular bone wall in element 11. (b) The root fracture and invasion of the biological space generated inflammation on the vestibular face.

After a CBCT scan, the diagnosis of vertical root fracture and invasion of the biological space with a compromised vestibular bone wall was confirmed (Figure 2(a)). The treatment proposed to the patient was an immediate dentoalveolar restoration with grafting material from the right maxillary tuberosity, implant, and immediate provision.

According to the protocol of the technique, the antibiotic therapy with amoxicillin 500 mg every 8 hrs was initiated five days before surgery and afterwards for another 7 days in order to reduce and modulate the inflammatory process aided by dexamethasone 4 mg 2 tablets 1 hour before surgery and 1 tablet every 8 hours for 3 days and paracetamol 750 mg every 8 hours for 3 days.

After an infiltrative anesthesia in the vestibule and the palatine region of tooth 11 with 2% mepivacaine and epinephrine 1 : 100,000 (DFL), an intrasulcular incision was made with a 15C (Swann-Morton) blade with the aid of a peritoneum, apical levers, and delicate forceps, minimally invasive exodontia was performed (Figure 2(b)) without compromising the integrity of the papillae. Thorough curettage and irrigation were performed until all granulation tissue was removed.

A mapping of the vestibular wall defect was performed using a millimeter probe inside the fresh alveolus and the milling sequence started at 5 mm from the cervical margin of the palatal bone wall and a cylindrical Cone Morse (Intraoss, Brazil) implant with a diameter of 4.0 mm × 13.0 mm to 3 mm infrabony, in a more palatinate position. Primary stability was achieved with a torque record of 35 Ncm. Altering the original technique advocated the preparation of the provisional crown at this time, using a titanium UCLA directly in the internal connection of the implant, and due to the objective of using an intermediate prosthetic abutment between the implant and the provisional crown, this phase was postponed until after bone reconstruction.

**Figure 3:**
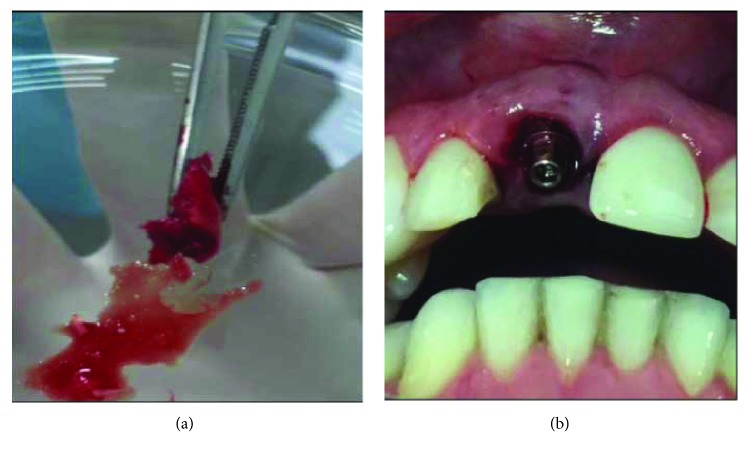
IDR surgical phase. (a) Cortical-medullary blade removed from the right maxillary tuberosity. (b) Cone Morse implant installation and pillar height test.

Anesthesia was performed in the region of the right tuft (2% mepivacaine, epinephrine 1 : 100,000) in the vestibular and ischemic anterior region at the crest and palatine area. A supracristal incision was performed until the periosteum and subsequent total displacement of the flap until the relaxing incisions in the region corresponding to the position where the third molar is generally located. With the aid of a surgical hammer and a goose chisel number 08, the cortical-medullary bone sheet was removed and taken to a glass plate where it was carved in the shape of the defect to be reconstructed (Figure 3(a)). The blade was then fitted into the defect, and the gap between the implant and the blade was filled with the remainder of the bone material collected from the tuberosity which was particulate with a delicate and properly condensed alveoli.

**Figure 4:**
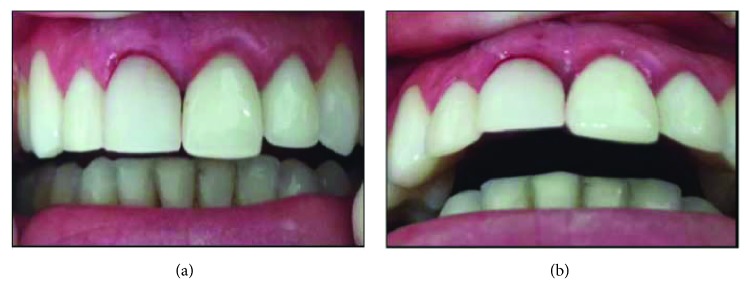
Immediate postoperative preserving the protective tissues with maintenance of vestibular volume and gingival margin. (a) Front view of the IDR. (b) Occlusal view with incisal relief.

The implant cover was removed and an intermediate pillar of 1.5 mm height was installed (Figure 3(b)). A stock tooth facet was captured under a titanium UCLA duly prepared with flow resin (Opallis, FGM) and Z-350 A1B composite resin (3M Espe). Adequate adjustments were made in the emergency profile and in the disocclusion guides with incisal height reduction. The reconstruction allowed the complete closure of the spaces and there was no need for sutures in tooth region 11 (Figures 4(a) and 4(b)). Only the tuft was adequately sutured with 4.0 silk thread (Ethicon).

Postoperative guidelines were followed in relation to a soft diet without using the tooth in question. The patient was monitored every 7 days, with total remission of the vestibular fistula.

**Figure 5:**
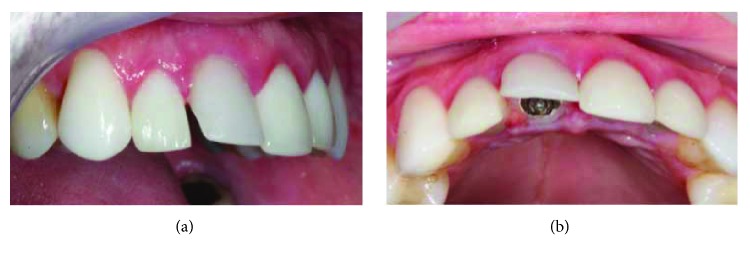
Trauma during sports procedure in the immediate implant region. (a) Due to the trauma, there was vestibularization. (b) Implant-crown turning compromising the entire osseointegration process.

Twenty-two days after the initial surgical procedure, the patient scheduled emergency care. He also reported that when doing a jiu-jitsu class, he received an elbow in the operated tooth with bleeding and pain after the trauma (Figures 5(a) and 5(b)).

**Figure 6:**
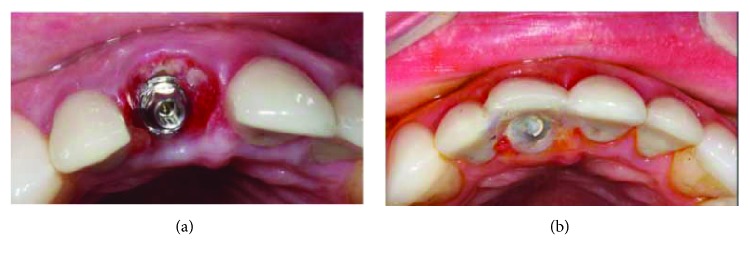
The provisional crown was removed and then reinstalled and attached to the neighboring teeth. (a) Presence of bone sequestration arising from the reconstruction of the vestibular wall. (b) Repositioning of the crown and union to the neighboring teeth with resinous cement.

After the clinical examination, a change was observed in the three-dimensional position of the implant/crown with vestibularization and gyroversion of the same. The crown was removed, and small fragments of bone abduction were found in the abutment region (Figure 6(a)). It was reinstalled and through the crown fixing screw, the entire structure was manipulated towards the palatal in an attempt to reposition it as close as possible to the original position. Temporary union with U-200 resin cement (3M Espe) of the acrylic crown was performed on the neighboring teeth 12 and 21 (Figure 6(b)). An implant tomography was ordered to assess the extent of the problem.

**Figure 7:**
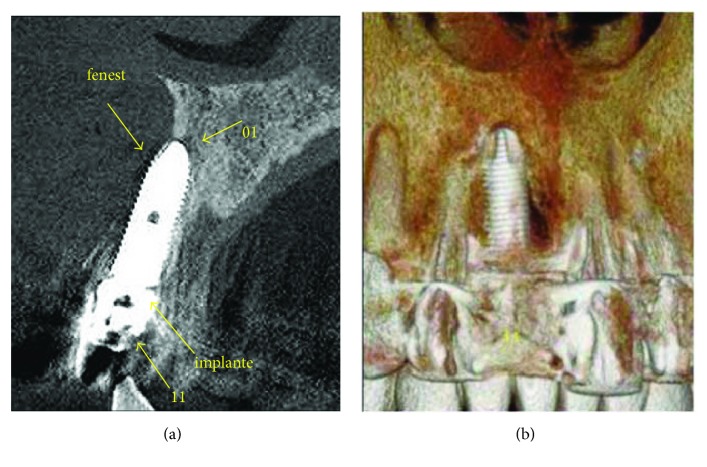
Radiographic analysis ten days after the trauma. (a) The tomographic image registers the implant in an inadequate three-dimensional position. (b) Loss of vestibular wall that had been rebuilt.

After ten days of trauma, the tomographic image analysis recorded that the three-dimensional positioning of the implant was out of the proper position, compromising the entire reconstruction of the vestibular wall. The loss of implant stability is directly related to the absence of supporting bone tissue in the vestibular area (Figures 7(a) and 7(b)).

Several treatment alternatives were then presented to the patient to remove the implant and to perform alveolar preservation through grafting of biomaterials and membranes for guided bone regeneration. However, it was a consensus among the surgeons that any of the cases presented could lead to a change and instability of the vestibular gingival margin, further compromising the esthetic outcome of the case. Alternatively, the possibility of retreatment through a new IDR was suggested. Although there were no reports of this, the idea behind the treatment was to consider the implant lost as if it were a root compromised with total loss of the vestibular bone wall, aided at this time by the absence of the infectious process.

With the endorsement of the patient, all the steps of the protocol of the technique were initiated through previous antibiotic therapy, and the surgical sequence was the same as previously described with only the following modifications: total curettage of the alveolus with removal of all the bone fragments and granulation tissue gift was performed; the new implant had a smaller diameter, 3.5 × 13 mm (Intraoss, Brazil), and its macrogeometry was conical; its insertion was searching the center of the alveolus, even if it was not possible to make a screwed prosthesis; and the maxillary tuberosity that provided the material for grafting was the left tuft.

**Figure 8:**
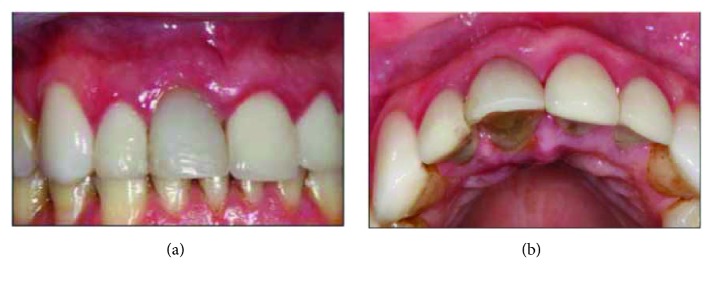
Immediate postoperative of the second IDR applied to element 11 with peri-implant tissue normality. (a) Immediate frontal view and (b) occlusal view demonstrating the maintenance of vestibular volume.

After implanting the implant with 45 Ncm torque, reconstruction of the buccal bone wall was performed with the insertion of medullary bone compacted at the apex of the implant up to half its length, only after the cortical-medullary lamina was positioned throughout the vestibular region leaving a 3 mm gap of the lamina to the implant that was filled by a new insertion of compacted medullary bone. A new 2.5 mm height abutment was installed with a torque of 32 Ncm, and immediate provisioning was still possible with a screwed crown even though the prosthetic connection of the abutment was in a more vestibularized position (Figures 8(a) and 8(b)).

**Figure 9:**
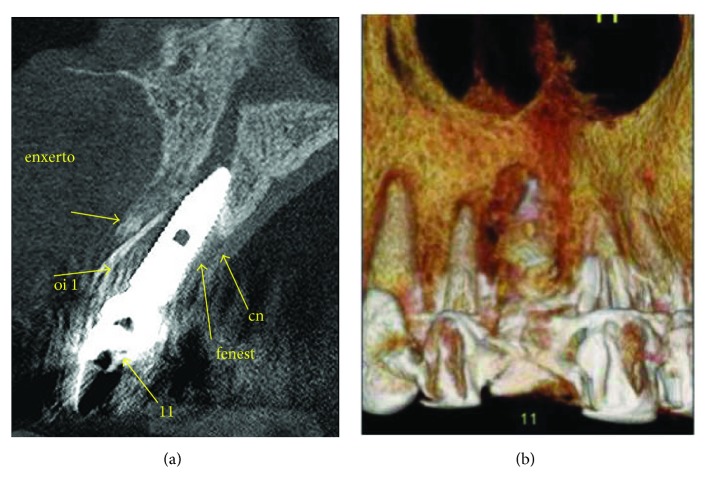
Tomography 10 days after surgery to visualize the 3D position of the implant and the presence of the cortical-medullar lamina. (a) Tomographic section where it is possible to visualize the grafted bone tissue. (b) Image recording bone reconstruction.

**Figure 10:**
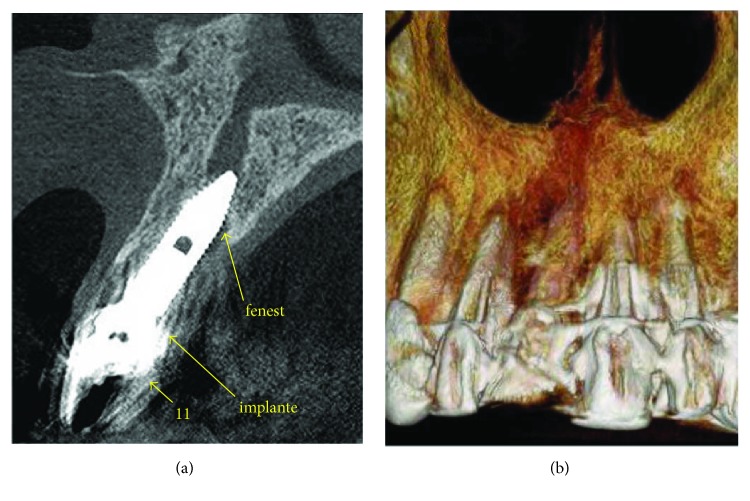
Tomography after five months of the second IDR. (a) Tomography performed with oral retractor to reveal soft tissues. (b) Recording of total graft incorporation and presence of a new vestibular bone wall.

The tubal suture was identical to the previous report, and the postoperative care was reinforced and amplified even with the use of an acrylic total plate in the upper arch to protect against any new trauma. The patient was monitored weekly until the ninety days of the surgical intervention and then every two weeks for a further two months. In this period, two CBCT scans were performed: the first ten days after the new IDR (Figures 9(a) and 9(b)) and the second after five months, confirming the osseointegration of the implant and maintenance of the new vestibular bone wall (Figures 10(a) and 10(b)).

**Figure 11:**
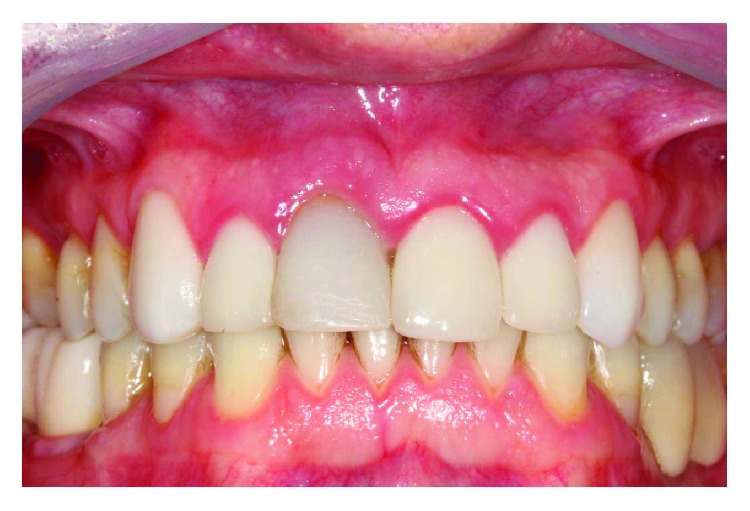
Result obtained after five months of healing. Despite clinical success, there was a slight discrepancy in the gingival margin of element 11.

In the sixth month after the new intervention, the screw crown was removed to evaluate the peri-implant soft tissue emergence and visualization profile. The pillar torque that continued with 32 Ncm was also verified. At this time, an esthetic evaluation of the gingival margin was made, and it was found that in the operated region, there was a smaller volume, and the gingival margin was more apical than the other incisors (Figure 11).

Even though he was satisfied with the result, the patient reported that he would like to improve his case as much as possible and that he would go through further interventions if necessary. Then, the possibility of a subepithelial connective tissue graft that would improve the height of the gingival margin and give more volume to the peri-implant tissues was presented to him, being of great value in the maintenance and stability of these tissues in the long term.

**Figure 12:**
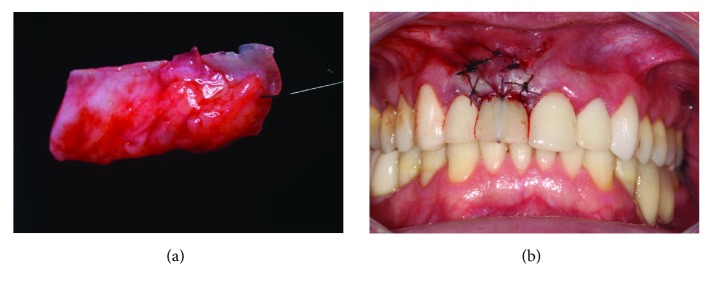
Connective tissue graft. (a) Subepithelial connective tissue removed from the palate. (b) Suture of the graft divided into the vestibular flap using the provisional crown as a foundation for better position and support of the gingival margin.

**Figure 13:**
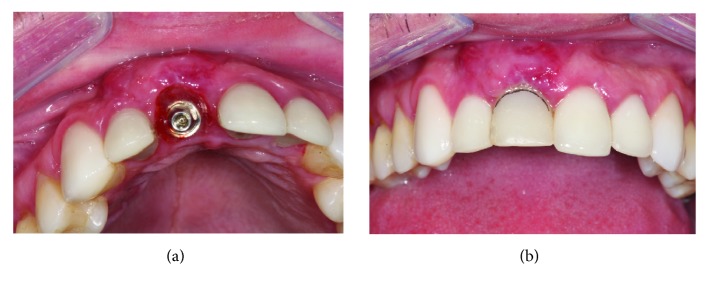
Grafted tissue in maturation process. (a) Occlusal view of the scar area after 30 days with increased volume. (b) Positioning of the new vestibular gingival margin.

**Figure 14:**
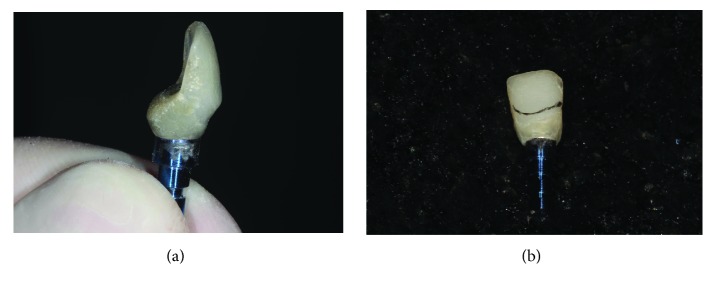
Correct delineation of the emergency profile is crucial to the long-term success of the technique. (a) Concave emergency profile in the cervicovestibular region. (b) The critical line corresponds to the position of the gingival margin, and below it is the subcritical area that should accommodate and maintain healthy peri-implant soft tissues.

After his endorsement, a new surgical procedure was scheduled. Anesthesia of the vestibular and ischemic fundus was performed in the vestibular region of tooth 11 and in the palatine region of teeth 14 to 16 with 3% articaine and epinephrine 1 : 100,000 (DLF). An intrasulcular incision with 15 C lamina (Swann-Morton) was performed around the implant, and the tissue division was done with the aid of tunneling. After mapping the amount to be grafted, the palate was accessed, and through an “L” incision, the connective tissue was removed (Figure 12(a)) and sutured in the vestibular region (Figure 12(b)) with 5.0 absorbable suture thread Technew. After the positioning of the graft with the flap moved to the incisor, the gingival margin was repositioned (Figures 13(a) and 13(b)), the crown was removed, the critical and subcritical area of the emergency profile was resized (Figures 14(a) and 14(b)), and the suspensory suture was made in the papillae and in the center of the margin and fixed directly to the acrylic crown with flow resin (Opallis, FGM) for better tissue stability. In the weeks following the removal of the points, the area of the emergency profile was checked and polished until complete visual healing of the peri-implant tissues.

**Figure 15:**
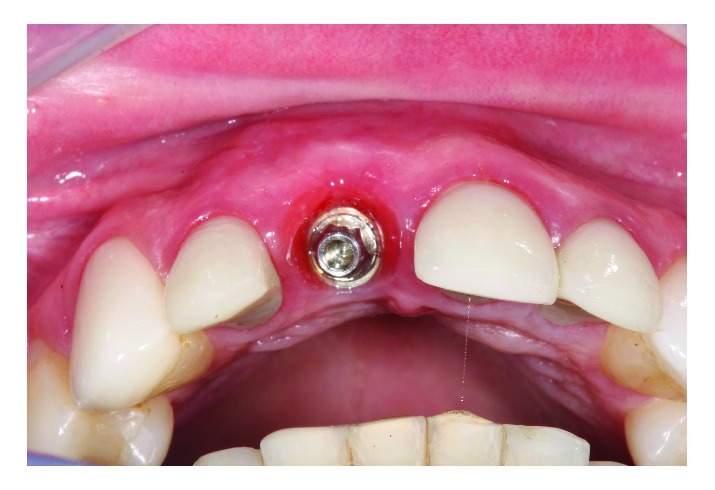
Occlusal view where the volume and quality of the peri-implant tissues obtained after the completion of the IDR with connective graft can be visualized.

Concluding this phase after ninety days, the provisional crown was removed, the final healing of the peri-implant tissues was evaluated, the pillar torque was checked, and the final crown was made (Figure 15).

The prosthetic phase begins with an additional silicone molding (Variotime, Heraeus/Kulzer) in an open tray with customization of the transfer abutment with acrylic resin (Pattern Resin LS, GC America Inc.) which copied the shape of the peri-implant tissues, achieved during the phase of the provisional crown.

After scanning of the model, an STL file was generated, and the analysis of the positioning of the implant/abutment complex was analyzed by the CAD of the Zirkonzahn system. It was decided by a cemented crown so that the technician had enough space to apply ceramic layers that reproduced the contralateral tooth in relation to shape and color.

**Figure 16:**
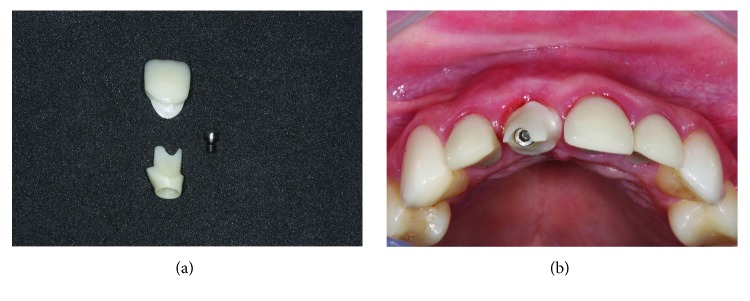
Prosthetic phase using custom zirconia abutment. (a) Screwed zirconia abutment and pure ceramic crown. (b) Occlusal view of the abutment screwed with the screw in the incisal line of the other incisors proving the more vestibular positioning of the prosthetic connection.

**Figure 17:**
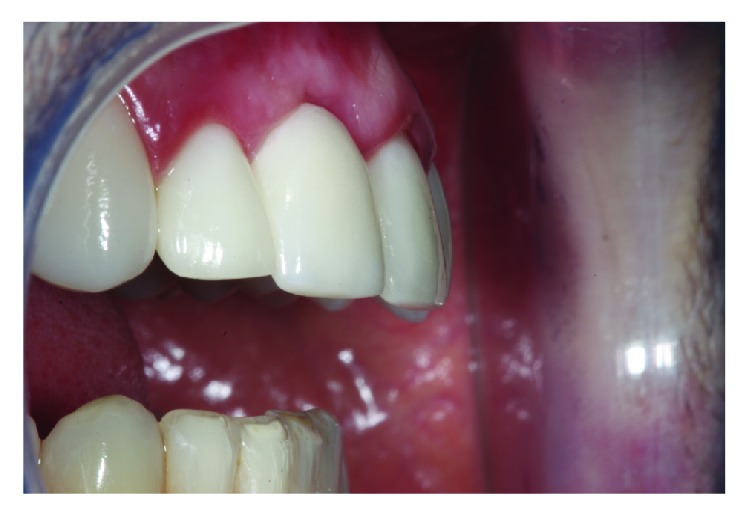
Lateral view of the abutment test and the stratified ceramic crown demonstrating adaptation to the peri-implant tissues, appropriate color, and shape.

**Figure 18:**
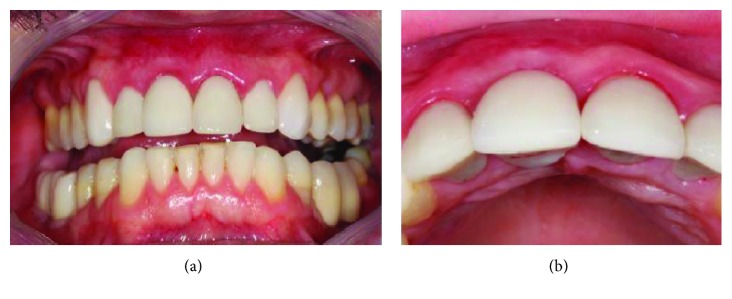
Final result after cementation with resin cement. (a) Front view of the cemented crown. (b) Occlusal view showing the correct 3D positioning of the prosthetic crown.

**Figure 19:**
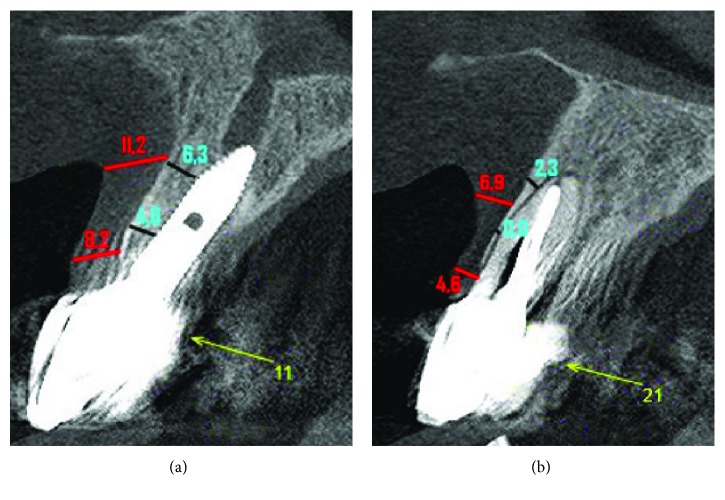
CBCT scan of the follow-up of the IDR reapplication. (a) Tomography with measures of bone and gingival tissue in the vestibule of implant 11 which was reconstructed by the surgical procedures. (b) Tomography where it is possible to compare the volumes of bone and gingival tissue in the vestibular area of a healthy tooth 21.

A custom screwed pillar and a coping, both using zirconia (Figures 16(a) and 16(b)), were milled (CAM, Zirkonzan). After the necessary tests and adjustments of the infrastructures, there was the application of stratified ceramics (Figure 17). The zirconia abutment was radiographed to confirm its adaptation and screwed with 32 Ncm torque, the screw hole was closed with Teflon tape (PTFE), the crown was cemented with U-200 resin cement (3M, Espe), and extreme attention was given to the cementation line so that there was no infiltration into the peri-implant tissues (Figure 18(a)). All occlusal adjustments were reviewed, and the disocclusion guides were checked (Figure 18(b)). A new tomography was performed with a buccal retractor in order to record the measurements of the hard and soft tissues in the vestibular region after twelve months of the IDR reapplication to determine the zero (follow-up zero) momentum, serving as a basis for follow-up and future comparisons (Figures 19(a) and 19(b)). Measurements at two points on the vestibular wall (cervical 4.6 mm and apical 6.3 mm at the implant) recorded the formation of the new vestibular wall with a considerable increase of the bone tissue, mainly in relation to the homologous tooth (21), whose measurements at similar points recorded a typical buccal bone board as described in the literature with values lower than 1 mm in the cervical and median and only in the apical region of the root with a measurement of 2.3 mm. In relation to the soft tissues, the subepithelial connective tissue graft practically doubled the values found in the contralateral tooth (8.2 mm versus 4.6 mm cervical, 11.2 mm versus 6.9 mm apical), being of paramount importance for maintaining a stable gingival margin and avoiding possible recessions that can affect the vestibular face of the immediate implants.

**Figure 20:**
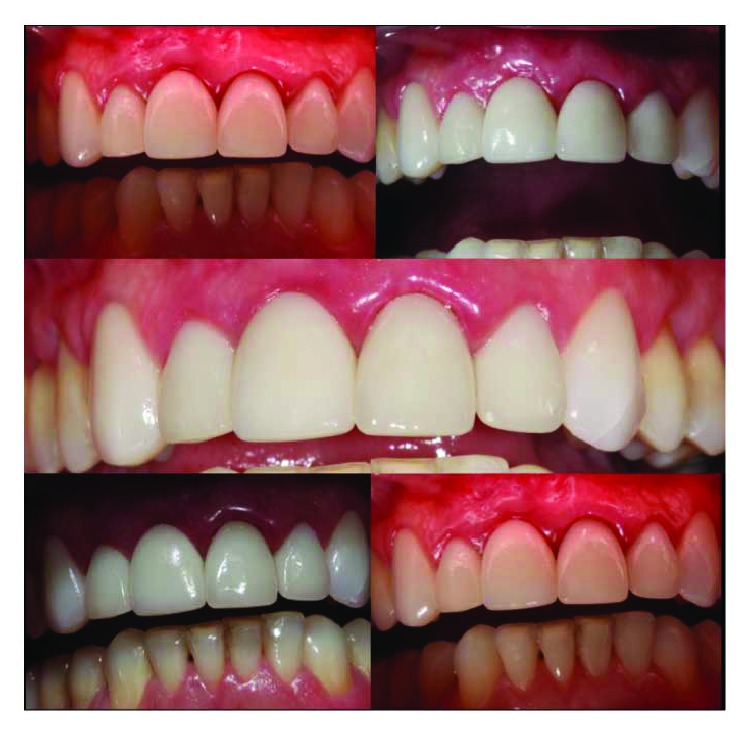
Case completed. Photo of the case with different light intensities, after one year of IDR reapplication, demonstrating the normality of the peri-implant tissues and the maintenance of the final gingival margin compatible with esthetic rehabilitation.

The end result in relation to the reconstruction of support and protection tissues projects clinical success in the long term (Figure 20).

## 3. Discussion

The literature describes that the buccal bone plate has on average less than 1 mm thickness and that the bone remodeling that occurs after the exodontia will alter the vestibular gingival margin independent of the installation of an immediate implant [8, 9].

Among the various methods to prevent spontaneous remodeling of tissues and to preserve the alveolar ridge, the most used is through biomaterials of low rate of reabsorption and protection with membranes, called guided bone regeneration (GBR) [10].

The major challenge for performing immediate implants is when one or more bone walls are lost. Clinically, the most common is the total or partial loss of the buccal bone wall, and IDR is presented as an alternative for the use of GBR. The idea of the technique is similar, and in the case of IDR, the cortical-medullary bone graft installed in the shape of the defect to be reconstructed will be a barrier to stabilize the particulate bone graft that will exist in the gap between the implant and the new bone wall. The use of the autogenous bone material of the maxillary tuberosity provides factors different from traditional biomaterials [11]. As the vascular pattern is vital for the success of bone grafts, the medullary nature of the grafts harvested at this site indicates that there is indeed a possibility of transferring bone material with viable and high-capacity osteoprogenitor cells into the receptor bed which provides faster and more effective healing with minimal alteration to the involved tissues. In addition to early and low-intensity stimulation that does not compromise mechanical stability, increased blood flow and contact osteogenesis will accelerate the full incorporation of the bone graft, ensuring the substrate necessary for the success of the implant and peri-implant tissues [12, 13].

The risks inherent in immediate loading techniques such as a minimally traumatic and flapless surgery, a 3D implant position, a gap filling, and its provisioning may not prevent postexodontic alveolar changes, and recessions may compromise the gingival margin and longevity of supporting and protective tissues [14].

The alveolus-dental topography in the anterior maxilla region, due to the inclination of the teeth, results in a very thin vestibular bone board and a thicker and more robust palatine bone wall [15]. The bone perforation for implant installation should always be in a palatal approach where greater primary stability is obtained. The preparation of the bone bed in this position allows the formation of a gap between the implant and the buccal wall that should be filled by grafting material. Its filling confers a substrate site for the osteoblasts to secrete a new bone matrix [16]. The materials most used for this purpose are autogenous bone and xenogenic hydroxyapatite. Both are important sources in reconstructive procedures and can often even be associated according to the need of the recipient site [17].

Proper positioning of the implant in the mesiodistal direction allows the preservation of the papilla, in the vestibular-palatine sense, determines the dimensions of the prosthetic crown, and is a preponderant factor in the choice of definitive restorations, whether cemented or screwed [18].

A vestibular wall approximately 2 mm thick is ideal for long-term esthetic success, as it allows peri-implant soft tissue stability. In cases of impairment of the buccal bone plate, the use of smaller-diameter implants concomitant with the regeneration of hard tissues will favor the maintenance of theses tissues [19, 20].

When mucosal deficiencies compromise planning and outcome, regenerative techniques should be associated to restore proper contour and volume. One of the measures is to optimize the presence of keratinized mucosa around the implants [21]. In these cases, it is recommended to perform a subepithelial connective tissue graft to prevent the vertical and horizontal volumetric loss of the peri-implant tissues. The presence of keratinized mucosa will provide texture and tone similar to natural teeth, hiding the tooth/crown interface and facilitating biofilm control [22–24].

The provisioning phase is one of the key factors for the immediate implants since it maintains the normal gingival architecture after the removal of a dental element [25]. The prosthetic crown loaded under the implant defines the shape of the gingival tissue relative to the bone crest and the mesial/distal contact points, the emergence profile of the provisional crown favors the maturation of the peri-implant tissues during the period of osseointegration of the implant, and the sculpture of the subcritical area determines the stability of the vestibular gingival margin [26, 27].

The analysis of the final tomography of this case shows that the IDR technique provided a significant increase of the vestibular bone wall when compared to the homologous tooth, whereas the healthy buccal bone of tooth 21 is less than 1 mm and the new vestibular bone wall of implant 11 is more than 2 mm. In addition to the fact that the subepithelial connective tissue graft created a new gingival biotype due to the increase in the volume and thickness of the peri-implant tissues, it can be affirmed that the necessary factors were created for the stabilization of the gingival margin in a position that did not compromise the esthetic results in the short and long term, according to the most recent studies described in the literature.

## 4. Conclusion

In spite of the intercurrences in the first phase of the IDR technique application, failure in osseointegration of the implant/bone graft complex allowed the clinical opportunity to measure the challenges of reapplying the IDR in an alveolus involved with the variables of the inflammatory events that occur with loss of an immediate implant. The result of success obtained significantly increases the possibilities of application of the technique and is of great assistance to the surgeon who, at the moment, is increasingly involved with the immediate implants in an esthetic area.
